# Minimal Antizyme Peptide Fully Functioning in the Binding and Inhibition of Ornithine Decarboxylase and Antizyme Inhibitor

**DOI:** 10.1371/journal.pone.0024366

**Published:** 2011-09-09

**Authors:** Ju-Yi Hsieh, Jung-Yen Yang, Chih-Li Lin, Guang-Yaw Liu, Hui-Chih Hung

**Affiliations:** 1 Department of Life Sciences and Institute of Bioinformatics, National Chung Hsing University, Taichung, Taiwan; 2 National Nano Device Laboratories and Department of Electrical Engineering, National Chiao Tung University, Hsinchu, Taiwan; 3 Institute of Medicine, Chung Shan Medical University, Taichung, Taiwan; 4 Institute of Microbiology & Immunology, Chung Shan Medical University, and Division of Allergy, Immunology, and Rheumatology, Chung Shan Medical University Hospital, Taichung, Taiwan; 5 Agricultural Biotechnology Center, National Chung Hsing University, Taichung, Taiwan; University of Delhi, India

## Abstract

Antizyme (AZ) is a protein with 228 amino acid residues that regulates ornithine decarboxylase (ODC) by binding to ODC and dissociating its homodimer, thus inhibiting its enzyme activity. Antizyme inhibitor (AZI) is homologous to ODC, but has a higher affinity than ODC for AZ. In this study, we quantified the biomolecular interactions between AZ and ODC as well as AZ and AZI to identify functional AZ peptides that could bind to ODC and AZI and inhibit their function as efficiently as the full-length AZ protein. For these AZ peptides, the inhibitory ability of AZ_95-228 was similar to that of AZ_WT. Furthermore, AZ_95-176 displayed an inhibition (IC_50_: 0.20 µM) similar to that of AZ-95-228 (IC_50_: 0.16 µM), even though a large segment spanning residues 177–228 was deleted. However, further deletion of AZ_95-176 from either the N-terminus or the C-terminus decreased its ability to inhibit ODC. The AZ_100-176 and AZ_95-169 peptides displayed a noteworthy decrease in ability to inhibit ODC, with IC_50_ values of 0.43 and 0.37 µM, respectively. The AZ_95-228, AZ_100-228 and AZ_95-176 peptides had IC_50_ values comparable to that of AZ_WT and formed AZ-ODC complexes with *K*_d,AZ-ODC_ values of 1.5, 5.3 and 5.6 µM, respectively. Importantly, our data also indicate that AZI can rescue AZ peptide-inhibited ODC enzyme activity and that it can bind to AZ peptides with a higher affinity than ODC. Together, these data suggest that these truncated AZ proteins retain their AZI-binding ability. Thus, we suggest that AZ_95-176 is the minimal AZ peptide that is fully functioning in the binding of ODC and AZI and inhibition of their function.

## Introduction

Polyamines such as putrescine, spermidine, and spermine can bind to DNA, RNA, and proteins to control a variety of physiological functions including DNA replication, gene regulation, cell cycling, apoptosis, post-translational modification and protein synthesis [1]–[3]. Elevated polyamine levels cause cell growth and differentiation in eukaryotes and thus may be highly associated with the development of cancer [2], [4]–[6]. Ornithine decarboxylase (ODC, EC 4.1.1.17) is a pyridoxal 5′-phosphate (PLP)-requiring enzyme that catalyzes the decarboxylation of L-ornithine to putrescine. ODC is the first and rate-limiting enzyme for polyamine biosynthesis [4], [7]–[8]. Due to the fact that elevated ODC activity increases polyamine levels in cells and that overexpression of ODC is associated with neoplastic transformation of cells [9]–[12], ODC is considered an oncogenic enzyme [7]. Therefore, inhibitors of ODC and the polyamine synthesis pathway have excellent therapeutic potential for many cancers [2], [5], [12]–[13].

Human ODC is a homodimer containing 461 amino acid residues with a molecular weight of 53 kDa per monomer [14]. ODC can be up-regulated by the c-*myc* and *ras* oncogenes [15] and is degraded within minutes through a process controlled by its regulatory protein, antizyme (AZ) [16]. The first mammalian AZ was discovered in 1976 [17]. Proteins are usually degraded through the ubiquitination pathway. However, ODC uniquely undergoes ubiquitin-independent degradation through non-covalent interactions with AZ [1], [16], [18]. The binding of AZ to ODC causes the dissociation of ODC dimers to produce AZ-ODC heterodimers, thus abolishing enzyme activity [7], [19]–[20]. Furthermore, the binding of AZ stimulates a conformational change in ODC that causes the enzyme to expose its C-terminal tail for recognition by 26S proteasome [21]–[23].

AZ was the first protein found to utilize translational frame shifting in the regulation of mammalian mRNA [19], [24]. Increased concentrations of polyamines induce the ribosome to bypass the first open reading frame (ORF) of AZ and allow the second ORF (+1 frame-shift) to synthesize a 228 amino acid residues with a molecular weight of 22-kDa, fully functional AZ protein [24]–[25]. AZ is regarded as a tumor suppressor gene because it inhibits ODC activity and polyamine transport and hinders many cancers caused by abnormal ODC and polyamine levels [1], [18]–[19], [26]–[28]. Additionally, the degradation of AZ is ubiquitin-dependent, and polyamine interferes with AZ degradation [29]–[30].

There are at least four AZ isozymes with different binding affinities for ODC [31]–[32]. Isoform 1, AZ-1, is present in all tissues and is the major isoform that participates in ODC degradation. The NMR structure of rat AZ-1 (residues 87–227) shows that it contains eight *β*-strands, *β*1–*β*8, and two *α*-helices, *α*1 and *α*2 [33]. The C-terminal region of AZ-1 interacts with ODC to inhibit enzyme activity, and the N-terminal region of AZ-1 controls the degradation of ODC [34]–[36]. AZ-2 has a distribution similar to that of AZ-1 in all of the tissues and cells examined thus far, but its expression level is significantly lower than that of AZ-1 [37]. Although AZ-2 can inhibit ODC enzyme activity, it does not promote ODC degradation, suggesting that it plays a role as a reversible storage compartment for stabilizing the ODC monomer by forming a heterodimer [38]–[39]. AZ-3 has tissue specificity in the germ cells of testes, where expression is restricted to the post-meiotic stage of spermatogenesis during the differentiation of male germ cells to mature sperm [40]. Male infertility has been linked to ODC overexpression in transgenic mice, implying that AZ-3 may play an important role in controlling ODC levels in male infertility. AZ-3 poorly inhibits ODC activity and fails to stimulate ODC degradation, but is similar to AZ-1 and AZ-2 in polyamine transportation [41]. AZ-4 has been found in the human brain and can also inhibit ODC activity, but its ability to stimulate ODC degradation and its role in polyamine uptake are still unknown [42].

Another protein that regulates ODC by binding to AZ is called antizyme inhibitor (AZI). AZI is homologous to ODC, but lacks ODC enzymatic activity [32], [43]–[46]. It binds to AZ with a higher affinity than ODC to sequester AZ from the AZ-ODC heterodimer and thus rescues ODC activity from AZ suppression and prevents rapid ODC degradation [20], [46]–[48]. AZI can bind to all isoforms of mammalian AZ [32], [42]. Overexpression of AZI increases cellular polyamine levels and thus results in cell proliferation and transformation [49]–[50], suggesting that AZI is also an oncogenic protein [46].

Because AZ appears to act as a tumor suppressor, it may be a good target for anticancer therapy. In this study, we focus on the biomolecular interactions between AZ and ODC as well as AZ and AZI to identify functional AZ peptides that could bind to ODC and AZI and inhibit their function as efficiently as the full-length AZ protein. Since many peptides, such as insulin, are used as drugs, fully functional AZ peptides may be used as candidates for anticancer therapy and these data may provide further information to guide drug designs of ODC and AZI.

## Results

### Inhibition of ODC enzyme activity by the AZ peptide

**Figure 1:**
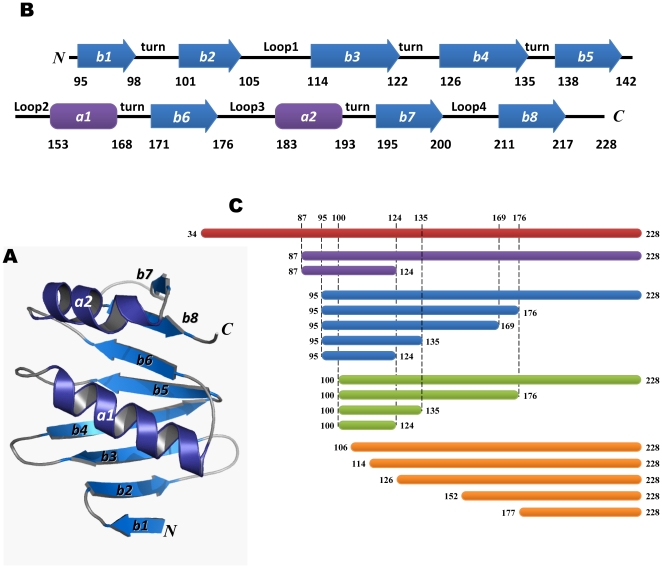
Homology model of human antizyme. (**A**) A homology model of human AZ using the NMR structure of rat AZ as the template [51]. (**B**) The secondary structure elements of human AZ from 95–228 consisting of two α-helices and eight β-strands. (**C**) A diagram of all truncated AZ peptides.

**Figure 2:**
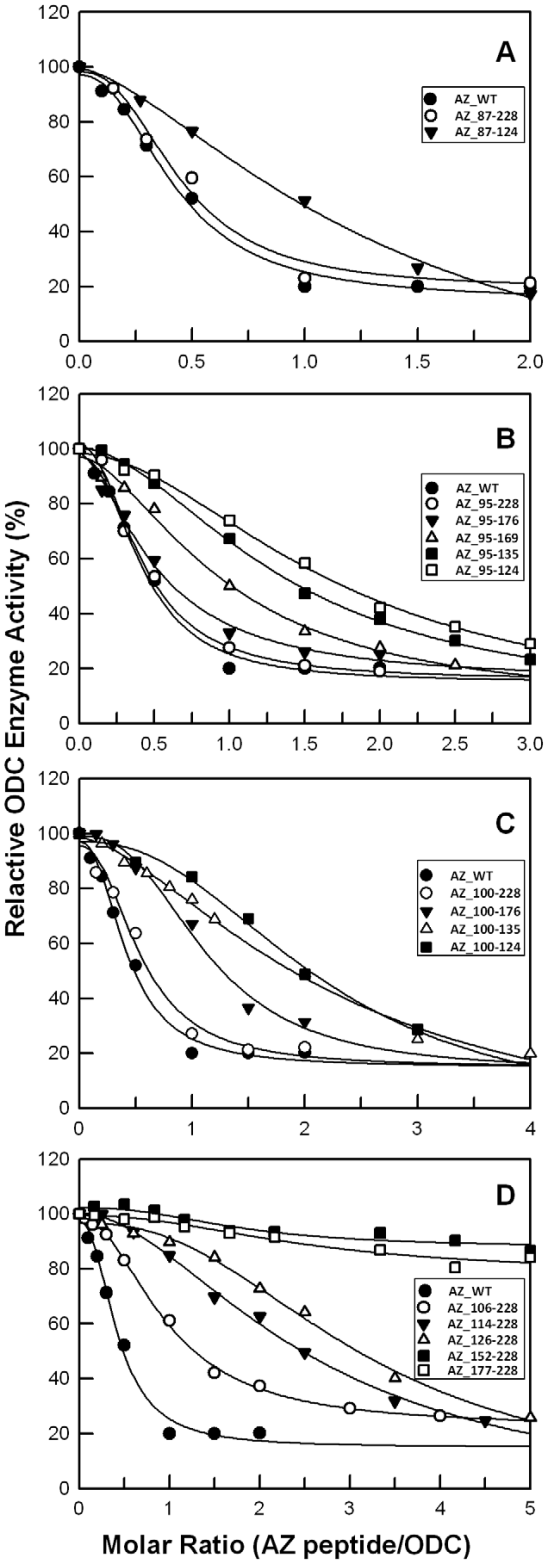
Relative ODC enzyme activity in the presence of AZ peptides. The activity of ODC was inhibited by various concentrations of AZ peptides. ODC concentrations were fixed at 20 µg/mL (0.19 µM). The IC_50_ values of AZ peptides shown in Table 1 were derived from fitting these inhibition curves. The molar ratio refers to AZ peptide versus ODC monomer.

A homology model of the human AZ structure was created using the NMR structure of mouse AZ as the template (Figure 1A, ESyPred3D: http://www.fundp.ac.be/sciences/biologie/urbm/bioinfo/esypred/) [51]. According to the secondary structure elements of AZ, we designed a series of AZ peptides to examine their ability to bind to ODC (Figures 1B and C). We first examined the inhibitory effect of these AZ peptides toward ODC enzyme activity (Figure 2) and calculated the IC_50_ values for these AZ peptides (Table 1).

**Table 1:** IC_50_ values for AZ_WT and peptides.

AZ Peptide	IC_50_ (µM) (without AZI)			IC_50 (AZ peptide)_/IC_50 (AZ_WT)_	IC_50_ (µM) (with AZI)			IC_50 (with AZI)_/IC_50 (without AZI)_
WT	0.16	±	0.02	1.0	0.50	±	0.003	3.1
87-228	0.17	±	0.02	1.1	ND			-
87-124	0.52	±	0.18	3.2	1.55	±	0.02	3.0
95-228	0.16	±	0.01	1.0	0.57	±	0.01	3.5
95-176	0.20	±	0.03	1.2	0.68	±	0.02	3.4
95-169	0.37	±	0.07	2.3	ND			-
95-135	0.51	±	0.03	3.2	1.59	±	0.02	3.1
95-124	0.65	±	0.11	4.0	1.98	±	0.02	3.0
100-228	0.21	±	0.04	1.3	0.62	±	0.01	3.0
100-176	0.43	±	0.08	2.6	1.42	±	0.02	3.3
100-135	0.86	±	0.19	5.3	ND			-
100-124	0.82	±	0.31	5.0	ND			-
106-228	0.37	±	0.02	2.3	ND			-
114-228	0.97	±	0.13	6.0	ND			-
126-228	1.19	±	0.17	7.3	ND			-
152-228	ND^a^			-	ND			-
177-228	ND			-	ND			-

All IC_50_ values with or without AZI were derived from fitting the inhibition curves of ODC shown in Figures 2 and 4, respectively.

a ND, not determined.

In the inhibition experiments, these AZ peptides displayed a differential inhibition ability that appeared to correlate with their length. The inhibition ability of the AZ_87-228 peptide was similar to that of AZ_WT but greater than that of AZ_87-124, indicating the importance of the C-terminus of AZ in binding to ODC (Figure 2A). The IC_50_ values for AZ_WT, AZ_87-228 and AZ_87-124 were 0.16, 0.17 and 0.52 µM, respectively. We further constructed AZ peptides from residue 95 to different ends of the C-terminus (95-series). The inhibition ability of AZ_95-228 was also similar to that of AZ_WT, and AZ_95-176 retained most of the inhibition ability, even though a large segment of 177–228 was deleted (Figure 2B). The IC_50_ values for AZ_95-228 and AZ_95-176 were similar to each other at 0.16 and 0.20 µM, respectively. However, a further deletion of AZ_95-176 from the C-terminus decreased the inhibition efficiency of AZ. The AZ_95-169 peptide, lacking *β*-strand 6 (*β*6), was only seven residues shorter than AZ_95-176, yet displayed a noteworthy decrease in ODC inhibition ability. The IC_50_ value of AZ_95-169 was 0.37 µM, 2.3-fold larger than that of AZ_WT. This indicates that *β*6 (residue 171–176) may contribute to ODC enzyme inhibition. Successive deletion of AZ_95-169 from C-terminus continued to reduce the inhibition ability of AZ. The IC_50_ values for AZ_95-135 and AZ_95-124 were 0.51 and 0.65 µM, respectively (Table 1).

Deletion from the N-terminus of AZ to different ends of the C-terminus was also performed (Figure 2C). AZ peptides from residue 100 (100-series) were constructed to compare with the 95-series of AZ peptides. The inhibition ability of AZ_100-228 was similar to that of AZ_WT and AZ_95-176 (Figures 2B and C); the IC_50_ value of AZ_100-228 was 0.21 µM. However, unlike AZ_95-176, which had an inhibition ability similar to that of AZ_95-228, the AZ_100-176 peptide had a lower inhibition ability than that of AZ_100-228 (Figure 2C). The IC_50_ value of AZ_100-176 was 0.43 µM, 2.6-fold larger than that of AZ_WT. Successive deletion of AZ_100-176 from C-terminus continued to reduce the inhibition ability of AZ. The AZ_100-135 and AZ_100-124 peptides displayed notably weaker inhibition ability than AZ_100-228 (Figure 2C). The IC_50_ values for AZ_100-135 and AZ_100-124 were 0.86 and 0.82 µM, respectively (Table 1).

The N-terminal truncation of AZ demonstrated relatively poor inhibition (Figure 2D). AZ_106-228 showed weaker inhibition ability than AZ_100-228 (Figures 2C and D), suggesting the importance of β-strand 2 (*β*2). Further deletion from the N-terminus to residue 113 (AZ_114-228) and to residue 125 (AZ_126-228) continued to impair the ability of AZ to inhibit ODC, as the IC_50_ values for AZ_106-228, AZ_114-228 and AZ_126-228 were 0.37, 0.97 and 1.19 µM, respectively. Although these AZ peptides had a reduced ability to inhibit ODC, they did lessen enzyme activity at high concentrations of AZ (Figure 2). In contrast, AZ_152-228 and AZ_177-228 showed almost no ODC inhibition, even at high concentrations of AZ (Figure 2D).

### Binding affinity of AZ peptides toward ODC

**Figure 3:**
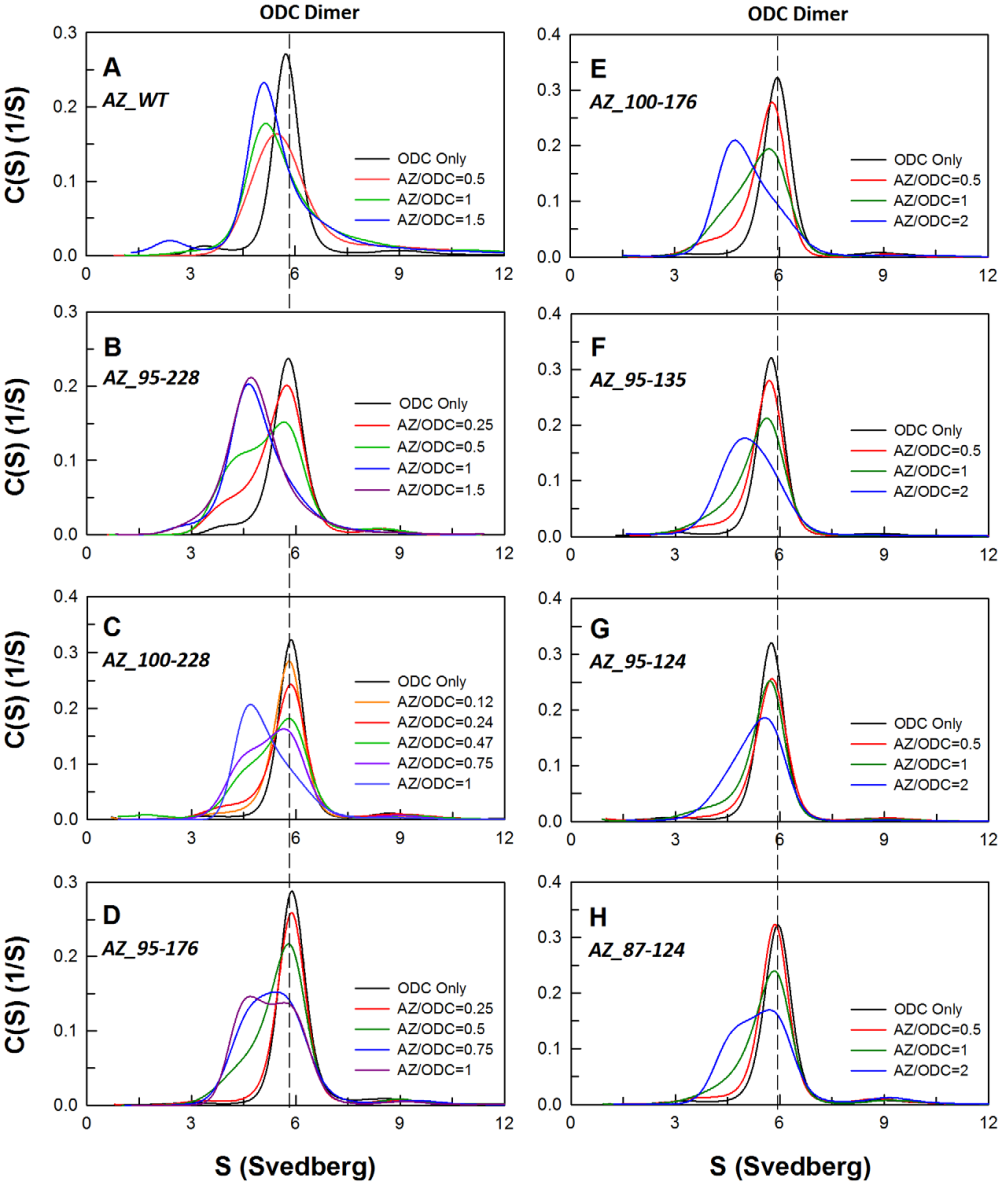
Continuous sedimentation coefficient distribution of human ODC in the presence of AZ peptide. The concentration of ODC was fixed at 0.3 mg/mL with concentrations of AZ ranging from 0.015 to 0.2 mg/mL (the molar ratio of AZ/ODC ranged from 0.12 to 2) in a buffer of 30 mM Tris-HCl (pH 7.4) and 25 mM NaCl at 20°C. The sedimentation velocity data were globally fitted with the SEDTHAT program to obtain *K*_d_ values for the AZ peptide-ODC complex (Table 2).

AZ binds ODC, dissociates the ODC dimer and then inactivates the enzyme [19], [20]. A size distribution analysis of ODC in the presence of AZ displayed the formation of AZ-ODC complexes (Figure 3A). In order to determine the differential binding affinity of these AZ peptides toward ODC, the size distributions of ODC with different concentrations of AZ peptides were analyzed. All sedimentation data were globally fitted with the AB hetero-association model in the SEDPHAT program to obtain the dissociation constant (*K*_d_) between ODC and AZ (Figure 3 and Table 2). AZ_WT disrupted dimeric ODC to form AZ-ODC complexes with a *K*_d,AZ-ODC_ value of 0.71 µM (Table 2). The AZ_95-228, AZ_100-228 and AZ_95-176 peptides all had IC_50_ values similar to that of AZ_WT and formed AZ-ODC complexes (Figures 3B–D, respectively) with *K*_d,AZ-ODC_ values of 1.5, 5.3 and 5.6 µM, respectively (2.1, 7.5 and 7.8-fold higher than that of AZ_WT). In contrast, the peptides that showed weaker inhibition, AZ_95-135, AZ_100-176, AZ_87-124 and AZ_95-124, formed AZ-ODC complexes (Figures 3E and F) with *K*_d,AZ-ODC_ values of 10.3, 10.8, 12.7 and 18.6 µM, respectively (14.5 to 26.2-fold higher than that of AZ_WT).

**Table 2:** Dissociation constants for human AZ-ODC and AZ-AZI complexes.

AZ peptide	*K*_d,AZ-ODC_ (µM)			*K*_d,AZ-AZI_ (µM)		
WT	0.71	±	0.005	0.027	±	0.0001
87-124	12.7	±	0.28	0.16	±	0.001
95-228	1.5	±	0.01	0.024	±	0.0002
95-176	5.6	±	0.04	0.06	±	0.004
95-135	10.3	±	0.07	ND		
95-124	18.6	±	0.13	0.28	±	0.002
100-228	5.3	±	0.03	0.068	±	0.0007
100-176	10.8	±	0.07	ND		

The dissociation constants (*K*_d_) of AZ-ODC and AZ-AZI were derived from global fitting of the sedimentation velocity data to the model of A+B↔AB hetero-association in the SEDTHAT program.

### Binding ability of the AZ peptide for AZI

**Figure 4:**
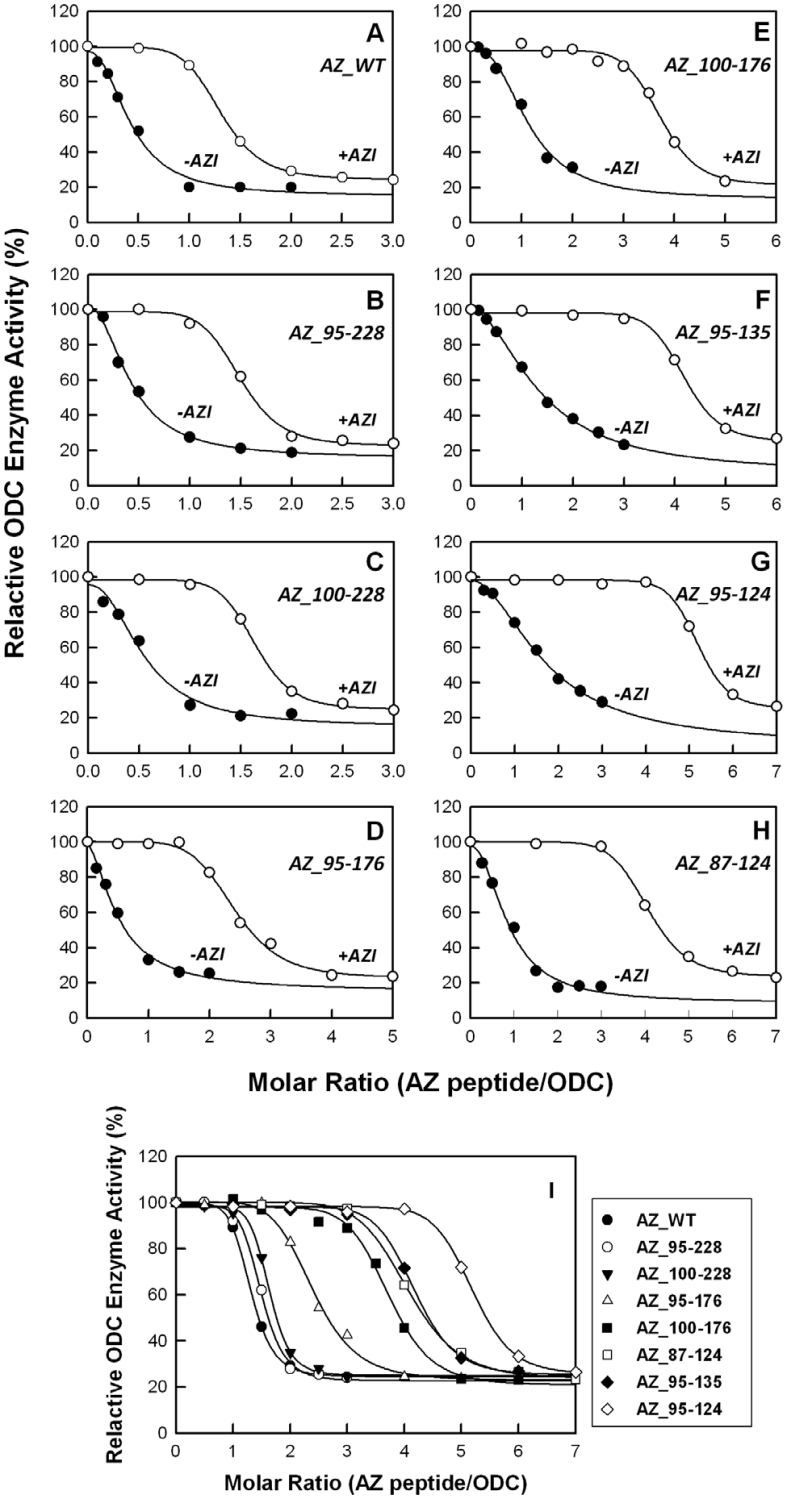
Relative ODC enzyme activities in the presence of AZ and AZI. ODC enzymes equilibrated with equimolar AZI were inhibited by various concentrations of AZ peptides. The concentrations of AZI and ODC were fixed at 18.9 µg/mL and 20 µg/mL, respectively (the ratio of AZI monomer versus ODC monomer was fixed at 1). **Panels A–H** show the inhibition curves of ODC for each AZ peptide in the absence (closed circles) and in the presence (open circles) of AZI. Panel I displays the inhibition curves in the presence of AZI for different AZ peptides.

AZ has a greater affinity for AZI than ODC, such that the formation of an AZ-AZI heterodimer will release the ODC monomer from an AZ-ODC complex and rapidly restore ODC enzyme activity [20]. Here, we examined the inhibition of ODC enzyme activity by AZ peptides in the presence of AZI. The ODC enzyme was first pre-incubated with AZI, keeping the molar ratio of AZI monomer/ODC monomer at unity. In the presence of AZI, ODC inhibition by AZ_WT and the AZ peptides was lower than in the absence of AZI; more AZ molecules were needed to inhibit ODC activity (Figure 4). Table 1 illustrates the IC_50_ values of the AZ peptides in the presence of AZI, showing that all of the IC_50_ values for the AZ peptides with AZI were larger than the values without AZI. The inhibition plots of the AZ peptides with AZI (Figure 4I) clearly demonstrate the differential inhibition ability among these AZ peptides. The IC_50 (with AZI)_ values of the AZ_95-228, AZ_100-228 and AZ_95-176 peptides, at 0.57, 0.62 and 0.68 µM, respectively, were similar to the IC_50 (with AZI)_ of AZ_WT. Furthermore, the IC_50 (with AZI)_ values of the weaker inhibitors, AZ_87-124, AZ_95-124, AZ_95-135 and AZ_100-176, were 1.55, 1.98, 1.59 and 1.42 µM, respectively. These data suggest that these AZ peptides still conserved their binding ability toward AZI. Interestingly, the ratio of IC_50 (with AZI)_/IC_50 (without AZI)_ for these AZ peptides remained constant around 3, implying the same relative binding preference for ODC and AZI.

To evaluate the binding affinity of these AZ peptides for AZI, the dissociation constants for AZ peptide-AZI were also determined (Supplemental Figure S1, Table 2). Because AZI has a higher affinity for AZ than ODC, the *K*_d_ values of these AZ-AZI complexes (*K*_d,AZ-AZI_) were smaller than those of the AZ-ODC complexes (*K*_d,AZ-ODC_). For AZ_WT, the *K*_d,AZ-AZI_ value of the AZ-AZI heterodimer was 0.027 µM. For the peptides that had *K*_d,AZ-ODC_ values similar to that of AZ_WT, AZ_95-228, AZ_100-228 and AZ_95-176, the *K*_d,AZ-AZI_ values were 0.024, 0.068 and 0.06 µM, respectively. The AZ peptides with weaker binding ability and a larger *K*_d,AZ-ODC_, AZ_87-124 and AZ_95-124, had *K*_d,AZ-AZI_ values of 0.16 and 0.28 µM, respectively.

## Discussion

AZ regulates ODC by binding to the enzyme, dissociating the ODC homodimer and inhibiting ODC enzyme activity. Previous studies of rat GST-AZ fusion protein have suggested that the C-terminal segment, GST-AZ_106-212, is important for the binding and inhibition of ODC [34]. Additionally, two functional regions in rat MBP-AZ fusion protein, amino acids 211–218 and 122–144, are considered to be important for the binding and inhibition of ODC [35]. We are the first group using biochemical and biophysical methods to demonstrate a minimal fully functional human AZ peptide, AZ_95-176, which is sufficient for both the inhibition of ODC enzyme activity and the binding of AZI. This human AZ peptide is stable without fusion to any protein and is shorter than those reported previously.

### AZ_95-176 is the minimal AZ peptide fully functional for binding and inhibition of ODC and AZI

Our data suggest that the N-terminus to amino acid 94 (1–94) is not required for AZ binding or inhibition of ODC because AZ_95-228 inhibits comparably to AZ_WT (Figure 2B, Table 2). Additionally, we found that AZ_95-176 and AZ_100-228 are fully functional and very similar to AZ_WT in binding and inhibition of ODC. The overlapping region of these two peptides is AZ_100-176, and we originally expected this fragment to be the minimal fully functional AZ peptide. However, the inhibition by AZ_100-176 was not as good as that of AZ_95-176 or AZ_100-228. The AZ_95-176 peptide comprises one *α*-helix and six *β*-strands, *α*1 and *β*1 to *β*6 while the AZ_100-228 peptide consists of *α*1, *α*2 and *β*2 to *β*8 (Figure 1B). For AZ_100-176, the lack of *β*1 may influence the structural conformation of the *β*-sheet, which may be required for AZ to interact with ODC. Although AZ_100-228 also lacks *β*1, the two additional *β*-strands, *β*7 and *β*8, may help to stabilize the structural conformation of the *β*-sheet, thus preserving its ability to bind to ODC.

Therefore, we suggest that the AZ_95-176 peptide is the minimal AZ peptide that is fully functional to bind and inhibit ODC. The impaired inhibition of peptides AZ_95-169 and AZ_100-176 support this inference. The deletion of amino acid 170 to 176 from the C-terminus (AZ_95-169) or the deletion of amino acid 95 to 99 from the N-terminus (AZ_100-176) in AZ_95-176 significantly decreases the ability of AZ in regards to the inhibition and binding of ODC. Additionally, the inability of AZ_177-228 to inhibit ODC activity also sustains this assumption. In the AZ_100-228 peptide, the segment from residue 177 to 228 (secondary structure elements *α*2, *β*7 and *β*8) is required for stability. However, this segment is not necessary for AZ functioning, and the essential elements of AZ are not located within this region because the AZ_177-228 peptide displays no inhibition of ODC activity (Figure 2D).

Furthermore, we propose that the key elements of AZ for binding AZI are the same as for ODC. AZI is homologous to ODC, although it can inactivate AZ function through the tighter binding of AZ, thus up-regulating ODC activity. Our data indicate that AZI can rescue AZ peptide-inhibited ODC enzyme activity (Figure 4). Furthermore, AZI can bind to AZ peptides with a higher affinity than ODC (Table 2). These data demonstrate that AZ-truncated proteins do not lose their AZI-binding ability, supporting the idea that the same elements of AZ are utilized to bind AZI and ODC. These AZ peptides may act as an antagonist against AZI for rescue of the ODC enzyme activity.

### The putative AZ-binding site of ODC and AZI is clasped by the essential elements of AZ within amino acids 95–176

**Figure 5:**
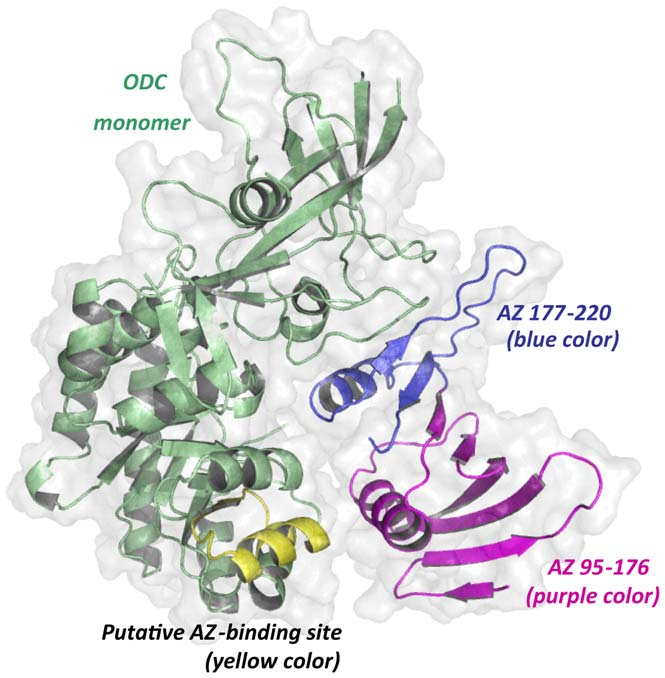
Docked structure of the mouse AZ-ODC complex. The molecular docking structure of the mouse AZ-ODC complex demonstrates a heterodimer [52] consisting of an ODC monomer and an AZ monomer. The putative AZ-binding site in ODC is colored yellow, and segment 95–176 of AZ is purple. This figure was generated using PyMOL (DeLano Scientific LLC, San Carlos, CA, USA).

Docked structures of mouse AZ-ODC and AZ-AZI complexes have been reported, and the docking results suggest that ODC and AZI occupy the same binding site on AZ [52]. These docked structures reveal that AZ binds within a large groove of ODC or AZI (Figure 5). In the complex structure, residues 95–176 of AZ face residues 117–140 of ODC, which is the putative AZ-binding site, thus supporting the role of segment 95–176 of AZ in the binding of ODC (Figure 5). However, a detailed structural view of the interactions between AZ and ODC remains to be elucidated.

### Anticancer therapy by blocking both ODC and AZI activity

Our study has shown that a series of AZ peptides displayed different efficiency in the binding of ODC and AZI and inhibition of their function. These AZ peptides, which are specific to ODC and AZI, may have low toxicity to cells and thus may have potential in developing pharmaceutical products for use in anticancer therapy. Further experiments regarding the effectiveness of these AZ peptides in depressing internal polyamines through the inhibition of polyamine biosynthesis and transport *in vivo* are needed in the future.

## Materials and Methods

### Expression and purification of recombinant proteins

Human wild-type ODC, AZ and AZI and a series of truncated AZ proteins were sub-cloned in the pQE30 vector (Qiagen) with an N-terminal His6-Tag sequence for further purification. The purification of these recombinant proteins were performed as described in Su *et al* [20]. Briefly, the expression vector was transformed into JM109 *Escherichia coli* (Stratagene). Protein overexpression in JM109 was induced with 1 mM isopropyl-1-thio-*β*-D-galactoside (IPTG) for 20 hr at 25°C. The crude extract was applied to a His-Select™ nickel affinity column (Sigma). The lysate-Ni-NTA mixture was washed using buffer containing 10 mM imidazole, 500 mM NaCl, 1 mM *β*-mercaptoethanol and 30 mM Tris-HCl at pH 7.6 to remove unwanted proteins. Finally, the target protein was eluted with elution buffer (250 mM imidazole, 500 mM NaCl, 30 mM Tris-HCl, and 1 mM *β*-mercaptoethanol, pH 7.6). Protein concentrations were determined by the Bradford method [53], and the protein purity was examined by sodium dodecyl sulfate polyacrylamide gel electrophoresis (SDS-PAGE).

### Construction of AZ truncation mutants

A QuikChange™ kit was used to generate the deletion of the AZ gene (Stratagene, La Jolla). The primer for the truncated mutant had to be at least 40 bases in length, with 15 bases on both deletion sides to complement the template DNA. The primer sequences for the truncations in the study were listed as follows:

87 (start) – 5′-GGATCGCATCACCATCACCATCACGATCACAATCTTTCAGC-3′;

95 (start) – 5′-GGATCGCATCACCATCACCATCACTTCTACTCCGATGATCG-3′;

100 (start) – 5′-GGATCGCATCACCATCACCATCACTTCTACTCCGATGATCG-3′;

106 (start) – 5′-GGATCGCATCACCATCACCATCACGAACTAACGTCCAACGAC-3′;

114 (start) – 5′-GGATCGCATCACCATCACCATCACAGGATTCTCAACGTCCAG-3′;

126 (start) – 5′-GGATCGCATCACCATCACCATCACAAACGCATTAACTGGCG-3′;

152 (start) – 5′-GGATCGCATCACCATCACCATCACAGCAAGGACAGCTTTGC-3′;

177 (start) – 5′-GGATCGCATCACCATCACCATCACCACAAGAACCGCGAGG-3′;

125 (stop) – 5′-CCAGGCTCACAGAC**TAG**AAACGCATTAACTGGCG-3′;

136 (stop) – 5′-CGAACAGTGCTGAGT**TAG**GGCAGCCTCTACATCG-3′;

170 (stop) – 5′-GGAGCAGCTGCGAGCC**TAG**CATGTCTTCATTTGC-3′;

177 (stop) –5′-GTCTTCATTTGCTTC**TAG**AAGAACCGCGAGG-3′. The mutant nucleotides are underlined and marked in bold. The polymerase chain reaction (PCR) was performed using *Pfu* DNA polymerase for a total of 16–18 cycles, and the product was then digested with DpnI to cleave the wild-type DNA template. Finally, the nicked DNA containing the desired mutations was transformed into the XL-1 *E. coli* strain, and the DNA sequence was verified by autosequencing.

### ODC enzyme activity assay

The ODC enzyme activity was determined using a CO_2_-L3K assay kit (DCL, Charlottetown, Canada) at 37°C. The continuous measurement of ODC enzyme activity was coupled to the carboxylation of PEP to oxaloacetate and the oxidation of oxaloacetate to malate, as previously reported [20]. The reaction mixture contains 30 mM Tris-HCl at pH 7.8, 10 mM ornithine, 0.2 mM pyridoxal 5′-pyrophosphate and 0.4 ml of the CO_2_-L3K assay kit solution, which contains 12.5 mM PEP, >0.4 U/ml phosphoenolpyruvate carboxylase (microbial), >4.1 U/ml malate dehydrogenase (mammalian) and 0.6 mM NADH analog in a final volume of 0.5 ml.

For the AZ inhibition experiment, the ODC enzyme (0.19 µM) and various amounts of AZ were added to the reaction mixture. The reaction was started after the ODC was added, and the absorbance decrease at 405 nm was continuously traced using a Perkin-Elmer Lamba-25 spectrophotometer. In this coupled reaction, the production of 1 mol of CO_2_ was concomitant with the oxidation of 1 mol of NADH analog. An extinction coefficient of 2410 m ^−1^ was used for the NADH analog in the calculations.

To evaluate the inhibitory effect of AZ, the inhibited ODC enzyme activity versus [AZ] was fitted with the following equation to estimate the IC_50_ value:
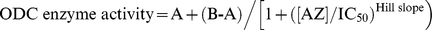
where A and B are the minimum and maximum ODC enzyme activity, respectively, and the Hill slope gives the largest slope of the curve. The IC_50_ value represents the concentration of AZ required for 50% inhibition of ODC enzyme activity. All of the calculations were done using the SigmaPlot 10.0 software program (Jandel, San Rafael, CA).

For enzyme rescue experiments with AZI, the ODC enzyme was first pre-incubated with AZ at a molar ratio of 3.5 AZ monomers to 1 ODC monomer to achieve 80% enzyme inhibition. The inhibited ODC activity was recovered by increasing the AZI concentration.

### Analysis of the AZ peptide-ODC complex size distribution by analytical ultracentrifugation

A Beckman Optima XL-A analytical ultracentrifuge was used for sedimentation velocity experiments. Reference and sample sectors in the centerpiece were filled with buffer (400 µl) and sample protein (0.3 mg/mL, 380 µl), respectively, and then the cell was built up in an An-50 Ti rotor. Sedimentation velocity experiments were performed at 20°C with a rotor speed of 42,000 rpm. The data were traced by absorbance at 280 nm in continuous mode, with a time interval of 420 s and a step size of 0.002 cm. A total of 20–30 scans at different time points were collected and fitted to a continuous size distribution model using the program SEDFIT [54]–[55]. All size distributions were calculated at 0.1 to 20 S with a confidence level of *p* = 0.95 and a resolution N of 200.

In order to determine the dissociation constants (*K*_d_) of the AZ peptide-ODC and AZ peptide-AZI complexes, sedimentation velocity experiments were performed at different concentrations of AZ or AZ peptide with a constant concentration of human ODC or AZI. The *K*_d_ values of the AZ peptide-ODC and AZ peptide-AZI complexes were calculated by a global fitting of all sedimentation data in the AB hetero-association model in the SEDPHAT program [56]–[57]. The partial specific volumes of the proteins, the solvent densities, and the viscosity were calculated by the SEDNTERP program [58].

## Supporting Information

Figure S1 **Continuous sedimentation coefficient distribution of human AZI in the presence of AZ peptide.** The concentration of AZI was fixed at 0.3 mg/mL with concentrations of AZ ranging from 0.05 to 0.24 mg/mL (the molar ratio of AZ/AZI ranged from 0.4 to 2.4) in a buffer of 30 mM Tris-HCl (pH 7.4) and 25 mM NaCl at 20°C. The sedimentation velocity data were globally fitted with the SEDTHAT program to obtain *K*_d_ values for the AZ peptide-AZI complex (Table 2).
(TIF)
