# Supplementary figures and images for: Minimal Antizyme Peptide Fully Functioning in the Binding and Inhibition of Ornithine Decarboxylase and Antizyme Inhibitor
Source: PLoS One. 2011 Sep 9;6(9):e24366. doi: 10.1371/journal.pone.0024366 (PMC3170320; doi:10.1371/journal.pone.0024366)

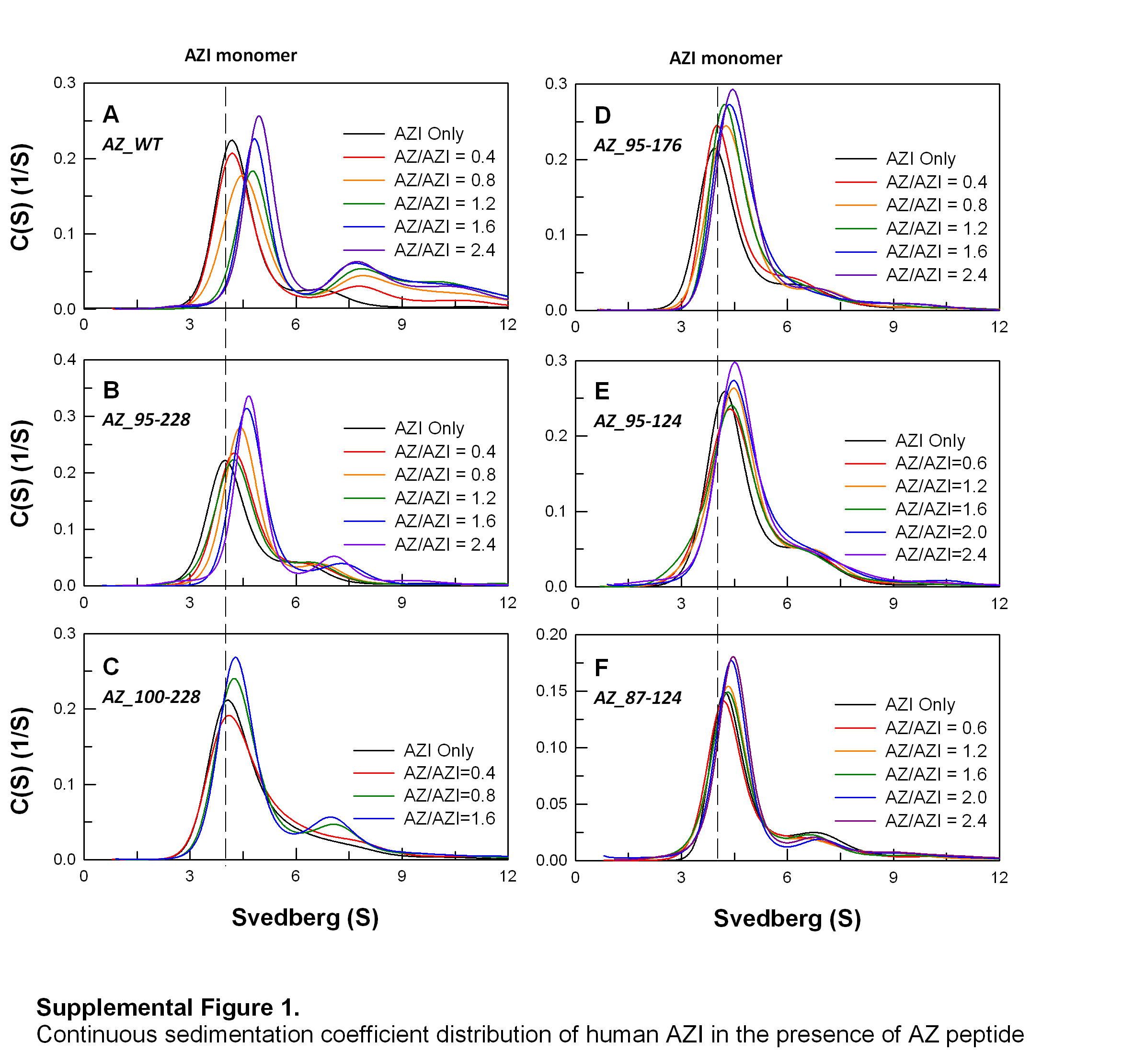

Supplement: Figure S1 — Continuous sedimentation coefficient distribution of human AZI in the presence of AZ peptide. The concentration of AZI was fixed at 0.3 mg/mL with concentrations of AZ ranging from 0.05 to 0.24 mg/mL (the molar ratio of AZ/AZI ranged from 0.4 to 2.4) in a buffer of 30 mM Tris-HCl (pH 7.4) and 25 mM NaCl at 20°C. The sedimentation velocity data were globally fitted with the SEDTHAT program to obtain K d values for the AZ peptide-AZI complex (Table 2). (TIF) [file pone.0024366.s001.tif]
